# Harnessing the power of artificial intelligence in predicting all-cause mortality in transcatheter aortic valve replacement: a systematic review and meta-analysis

**DOI:** 10.3389/fcvm.2024.1343210

**Published:** 2024-05-31

**Authors:** Faizus Sazzad, Ashlynn Ai Li Ler, Mohammad Shaheryar Furqan, Linus Kai Zhe Tan, Hwa Liang Leo, Ivandito Kuntjoro, Edgar Tay, Theo Kofidis

**Affiliations:** ^1^ Department of Surgery, Yong Loo Lin School of Medicine, National University of Singapore, Singapore, Singapore; ^2^ Department of Biomedical Informatics, Yong Loo Lin School of Medicine, National University of Singapore, Singapore, Singapore; ^3^ Department of Biomedical Engineering, College of Design and Engineering, National University of Singapore, Singapore, Singapore; ^4^ Department of Cardiology, National University Heart Centre, Singapore, National University Hospital, Singapore, Singapore; ^5^ Asian Heart & Vascular Centre (AHVC), Mount Elizabeth Medical Centre, Singapore, Singapore

**Keywords:** aortic valve replacement, transcatheter, systematic review, transcatheter aortic valve prosthesis, mortality, artificial intelligence, machine learning

## Abstract

**Objectives:**

In recent years, the use of artificial intelligence (AI) models to generate individualised risk assessments and predict patient outcomes post-Transcatheter Aortic Valve Implantation (TAVI) has been a topic of increasing relevance in literature. This study aims to evaluate the predictive accuracy of AI algorithms in forecasting post-TAVI mortality as compared to traditional risk scores.

**Methods:**

Following the Preferred Reporting Items for Systematic Reviews and Meta-analyses for Systematic Reviews (PRISMA) standard, a systematic review was carried out. We searched four databases in total—PubMed, Medline, Embase, and Cochrane—from 19 June 2023–24 June, 2023.

**Results:**

From 2,239 identified records, 1,504 duplicates were removed, 735 manuscripts were screened, and 10 studies were included in our review. Our pooled analysis of 5 studies and 9,398 patients revealed a significantly higher mean area under curve (AUC) associated with AI mortality predictions than traditional score predictions (MD: −0.16, CI: −0.22 to −0.10, *p *< 0.00001). Subgroup analyses of 30-day mortality (MD: −0.08, CI: −0.13 to −0.03, *p* = 0.001) and 1-year mortality (MD: −0.18, CI: −0.27 to −0.10, *p *< 0.0001) also showed significantly higher mean AUC with AI predictions than traditional score predictions. Pooled mean AUC of all 10 studies and 22,933 patients was 0.79 [0.73, 0.85].

**Conclusion:**

AI models have a higher predictive accuracy as compared to traditional risk scores in predicting post-TAVI mortality. Overall, this review demonstrates the potential of AI in achieving personalised risk assessment in TAVI patients.

**Registration and protocol:**

This systematic review and meta-analysis was registered under the International Prospective Register of Systematic Reviews (PROSPERO), under the registration name “All-Cause Mortality in Transcatheter Aortic Valve Replacement Assessed by Artificial Intelligence” and registration number CRD42023437705. A review protocol was not prepared. There were no amendments to the information provided at registration.

**Systematic Review Registration:**

https://www.crd.york.ac.uk/, PROSPERO (CRD42023437705).

## Introduction

Transcatheter aortic valve implantation (TAVI) is a crucial procedure in treating severe aortic stenosis, which is characterised by the narrowing of the aortic valve (1). TAVI provides a minimally invasive alternative to open-heart surgery for older patients and those with numerous comorbidities (2) with faster recovery times, shorter hospital stays, and less procedural risks (3–6). In patients with severe aortic stenosis, TAVI has been found to reduce symptoms, as well as increase quality of life and overall survival rates (7, 8). The emphasis of this paper is to predict the risk for TAVI, given that it has already been proven to have multiple benefits.

The mortality rate after TAVI might vary depending on a number of factors, including patient characteristics, comorbidities, and intra-procedural concerns. For intermediate-risk patients undergoing TAVI, longer-term follow-up in the PARTNER 2 study revealed a death rate of 26.2% at 5 years (9, 10). The SURTAVI trial showed that all-cause mortality was 31.3% at 5 years using the CoreValve self-expanding prosthesis in intermediate-risk patients (11). In a similar vein, the CoreValve U.S. Pivotal High-Risk Study found that high-risk patients had a 5-year mortality rate of 47.9% (12), and a separate study found the mortality rate to be 58.8% (13). The NOTION trial demonstrated that in low-risk patients, the mortality rate of TAVI with the first-generation CoreValve self-expanding prosthesis was 27.6% after 5 years of follow up (14). The PARTNER 3 trial also showed that the 5-year all-cause mortality for TAVI was 10.0% (15). One of the more recent trials, Evolut trial, found that the 4-year mortality for TAVI using self-expanding CoreValve was 10.7% (16). A separate meta-analysis has also found the all-cause mortality to be higher in low-risk patients undergoing TAVI over surgical aortic valve replacement (27.5% vs. 17.3%) (17). Thus, given the high probability of fatality and differences in mortality depending on the risk levels of patients, prediction prior to the start of the procedure is increasingly necessary.

Improving risk assessment and patient outcomes may be possible with the application of artificial intelligence (AI) in predicting death after TAVI. In-depth patient data analysis by AI algorithms has the ability to uncover pertinent trends and risk factors that can help forecast post-TAVI death (18). These models can help identify high-risk individuals who might need more measures or more intensive postoperative surveillance. On the other hand, AI models could also identify patients who may not benefit from TAVI, given that the risk of mortality outweighs the benefits of undergoing the procedure.

Thus, our goal in analysing the accuracy of AI-generated death forecasts in TAVI procedures was to determine how well AI algorithms can predict mortality outcomes for patients undergoing TAVI. This evaluation aims to assess the effectiveness of AI models in predicting post-TAVI death rates and evaluate the accuracy of these forecasts.

## Methods

We conducted a systematic review following guidelines from the Preferred Reporting Items for Systematic Reviews and Meta-analyses for systematic review (PRISMA) standard (19). In total, we performed our search on 4 databases, including PubMed, Medline, Embase and Cochrane, from the date of inception to 24 October 2023. Across all databases, combinations of different search terms, including Medical Subject Headings (MeSH) terms were generated. The following search strings were used: “Transcatheter aortic valve replacement AND artificial intelligence”, “Transcatheter aortic valve replacement AND machine learning”, “Transcatheter aortic valve replacement AND deep learning”, “Transcatheter aortic valve implantation AND artificial intelligence”, “Transcatheter aortic valve implantation AND machine learning”, “Transcatheter aortic valve implantation AND deep learning”, “Aortic stenosis AND artificial intelligence”, “Aortic Stenosis AND machine learning” and “Aortic stenosis AND deep learning”. The search was perfomed using MeSH terms only.

### Inclusion and exclusion criteria

Any retrospective studies that reported the use of AI to predict post-operative mortality in TAVI patients were included in our analysis. There were no randomised controlled trials identified in our search. We excluded articles that used AI to predict aortic stenosis, intra-cardiac parameters such as aortic valve annulus, studies on heart murmurs, hypertrophic cardiomyopathy, paediatric studies, narrative articles and conference abstracts. We also excluded studies on complicated TAVI patients (e.g., TAVI with infective endocarditis or cancer) and articles that used AI to predict other post-TAVI parameters such as cerebrovascular complications, pacemaker implantation, heart failure, readmission, length of hospital stay and bleeding.

### Study selection

To determine suitability for inclusion of each study, we first assessed the studies by their titles and abstracts and then retrieved the full-text records should the study either fulfil the inclusion criteria or if the reviewer was uncertain of the article's suitability. In order to ensure reproducibility of our study selection, the studies were independently screened and evaluated by three reviewers. All disagreements were solved by consensus amoung the reviewers with no modification of the search and inclusion criteria.

### Quality of evidence and risk of bias

GradePro quality of evidence assessment software was used to evaluate the included studies as illustrated in the Cochrane handbook of reviews (20). The risk of bias in all observational cohort studies was also assessed according to guidelines from the Cochrane handbook. Risk of bias was evaluated using the Risk of Bias in Non-randomised Studies of Interventions (ROBINS-I) tool (21).

### Outcomes of interest

Data from each article was collected by two authors (AL, LT). The following variables were abstracted for analysis: Authors, year of publication, study type, patient sample size, post-TAVI mortality, age, female, AI algorithms used. Our primary outcome measure was the area under curve (AUC) value [mean and 95% confidence interval (CI)] of AI models predicting post-TAVI all-cause mortality. We included data on intra-hospital mortality, 30-day mortality, 1-year mortality and 5-year mortality in our analyses. For studies that reported AUC values of both an internal and external validation cohort, data from the latter was abstracted for analysis.

### Statistical analysis

Studies that reported data comparing AI and traditional scores were analysed using Review Manager 5.3 (RevMan 5.3) software (22). Due to the limitations of RevMan 5.3 in pooling AUC values, reported means were converted to negative values and inputted into the software. We calculated the mean difference (MD) as the outcome effect measure used in our double-arm meta-analysis. For studies that reported 95% CI, the in-built RevMan calculator was used to estimate the missing standard deviations (SD). For single-arm meta-analysis, the STATA 17 software was used to pool study data and generate forest plots (23). In order to adjust for statistical heterogeneity across all study populations, all data was analysed using random effects models. All data is reported in mean and 95% CI [lower limit, upper limit]. In our subgroup analysis, we divided the studies by intra-hospital, 30-day, 1-year and 5-year mortality. For the 1-year mortality subgroup, gradient boosting and extreme gradient boosting models were grouped together as the models are similar and we had insufficient data to compare each individually. Similarly, non-gradient-boosting algorithms (random forest, decision tree, artificial neural network, multilayer perceptron) were also grouped together for subgroup analysis.

#### AI algorithms

The AI algorithms of interest to this study included random forest (RF), gradient boosting (GB), artificial neural network (ANN), multilayer perceptron (MLP) and logistic regression (LR). This section includes short descriptions of each algorithm. Further elaboration on the pros and cons of each algorithm can be found in the discussion section.

The RF model constructs a series of decision trees and utilises bagging and randomisation of predictors in order to accurately predict outcomes (24). The GB model involves an ensemble of weak learner decision trees. Each weak learner progresses through a stepwise progression, where previous iterations of weak learners are combined into a successive strong learner, correcting for the errors of the preceding learner each time (25, 26). The ANN algorithm is a mathematical model developed based on the transmission of signals amoung neural networks in biological nervous systems. An input signal is channelled through a collection of nodes, which are artificial neurons (27). Each node analyses the input signal and then transmits an output to each of its connected neurons, mimicking the transmission of action potentials across neurons and synapses in the human brain. The nodes are also organised into layers. The input signal travels from the first input layer to the last output layer, undergoing different transformations at each layer. The MLP algorithm is a class of feedforward ANN that is comprised of an input layer, one or more hidden layers and an output layer. The LR algorithm fits a logistic function onto a dataset and predicts the probability of an independent variable, such as mortality, from a dependent variable. The maximimum likelihood estimation method is most commonly used to maximise the likelihood function and find the optimal fit of the model (28).

## Results

**Figure 1:**
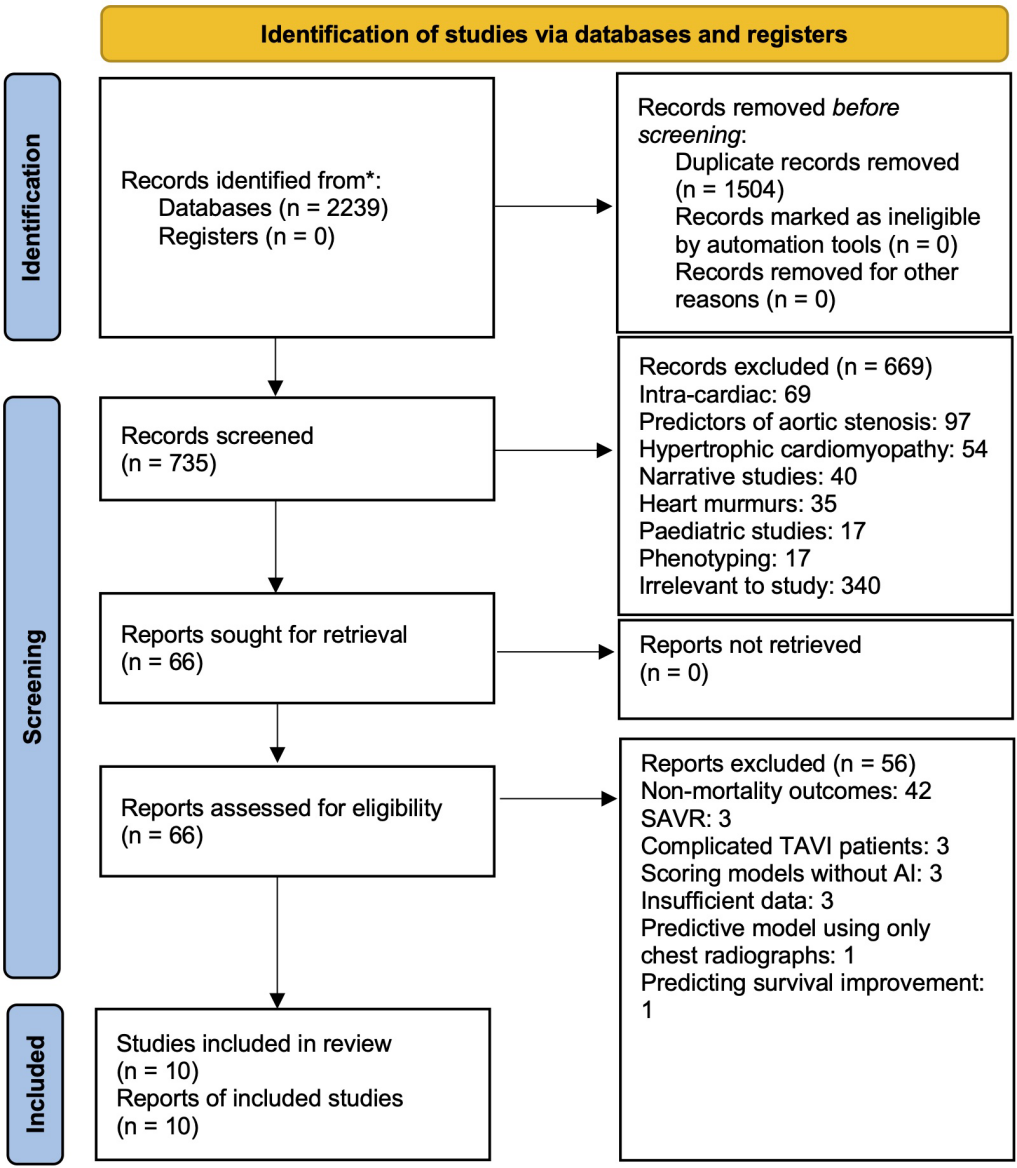
PRISMA flow chart showing systematic search. 2,234 articles were discovered on initial search, 738 remained after duplicates were removed. After our exclusion criteria was applied to record screening based on title, abstract and full-text assessment, 10 articles remained for inclusion in our analysis.

In total, our systematic search yielded 2,239 records. After 1,504 duplicate records were removed, 735 remained for title and abstract review. The high number of duplicates was likely due to our search strings throughout the various databases producing similar articles. Based on our exclusion criteria, we eliminated 669 studies and retrieved full-text articles for 66 articles. Finally, a further 56 articles were excluded based on full-text assessment, leaving 10 studies (29–38) for data abstraction (Figure 1).

### Risk of bias assessment

We conducted a risk of bias assessment on the included studies. Overall, all 10 studies were retrospective cohort studies and were thus prone to the inherent bias associated with observational study designs. No serious risk of bias was detected in any of the included studies (Table 1).

**Table 1:** Summary of studies and gradePro quality of evidence.

Study	Study design	Risk of bias	Inconsistency	Indirectness	Imprecision	Patients (n)	Mortality (%)	Risk score (%)	Age (years)	Female (%)	AI algorithms used
Agasthi et al. (29)	R/O	Not serious	Not serious	Not serious	Not serious	1,055	14	STS score	80.9 ± 8.0	42	GB
								7.9 ± 5.1 (Alive)			
								9.7 ± 5.3 (Deceased)			
Gomes et al. (30)	R/O	Not serious	Not serious	Not serious	Not serious	451	5.5	STS score 5.1 ± 3.4	82.7 ± 5.7	51.7	ANN, RF, support vector machine
Hernandez-Suarez et al. (31)	R/O	Not serious	Not serious	Not serious	Not serious	10,883	3.6	-	81.0 ± 8.5	47.7	ANN, RF, LR, Naive Bayes
Kwiecinski et al. (32)	R/O	Not serious	Not serious	Not serious	Not serious	823	12	EuroScoreII	82.0 ± 5.0	54	GB
								4.8 [3.0–6.3] (Derivation cohort)			
								5.1 [3.3–7.4] (Validation cohort: Medical University of Warsaw)			
								4.5 [2.9–7.3] (Validation cohort: Hospital Clinico San Carlos)			
Leha et al. (33)	R/O	Not serious	Not serious	Not serious	Not serious	22,283 (Cross-validation)	3.3	STS score	81.0 ± 6.1	53.2	RF
								5.9 ± 4.6			
						5,864 (Generalisation)					
Lertsanguansinchai et al. (34)	R/O	Not serious	Not serious	Not serious	Not serious	178	4.1	STS score	81.6 ± 8.3	56.2	Decision tree
								7.79 ± 5.51			
Lopes et al. (35)	R/O	Not serious	Not serious	Not serious	Not serious	1,160 (Centre A)	10	-	81.0 ± 7.0 (Centre A Survived, *n* = 1,039)	44 (Centre A survived, *n* = 1,039)	ANN (narrow and wide), RF, GB Catboost
						631 (Centre B)					
Mamprin, Lopes et al. (36)	R/O	Not serious	Not serious	Not serious	Not serious	1,300 (AMC centre)	9.9	-	81.0 ± 7.0 (AMC Survived, N = 1,171)	56 (AMC Survived, *N* = 1,171)	RF, LR, Catboost
						631 (CZE-TU centre)					
Mamprin, Zelis et al. (37)	R/O	Not serious	Not serious	Not serious	Not serious	270	11.1	-	80.7 ± 6.2	48	GB
Penso et al. (38)	R/O	Not serious	Not serious	Not serious	Not serious	471	45	EuroScoreII	81.0 ± 6.0	63.7	ANN (MLP), RF, GB, LR
								16 (10–21)			

R/O, retrospective/observational; AMC, Amsterdam University Medical Centre; ANN, artificial neural network; CZE-TU, collaboration between Catharina Ziekenhuis Eindhoven and Technische Universiteit Eindhoven; GB, gradient boosting; LR, logistic regression; MLP, multilayer perceptron; RF, Random forest.

### Summary of included studies

Of the 10 included studies, 6 (30, 31, 33–36) reported data on the RF algorithm, 4 (29, 32, 35, 37) on GB, 4 (30, 31, 35, 38) on ANN and MLP, and 3 (31, 36, 38) on LR. 5 studies (30, 31, 35, 36, 38) reported using more than 1 AI model for mortality prediction. The traditional scores used in the studies included the STS score (30, 33), TAVI_2_-SCORE (29), EuroSCORE II (32, 38) and CoreValve score (34). A summary of the predictive variables used in each study is provided in Supplementary Table S1.

### Use of AI algorithms

**Figure 2:**
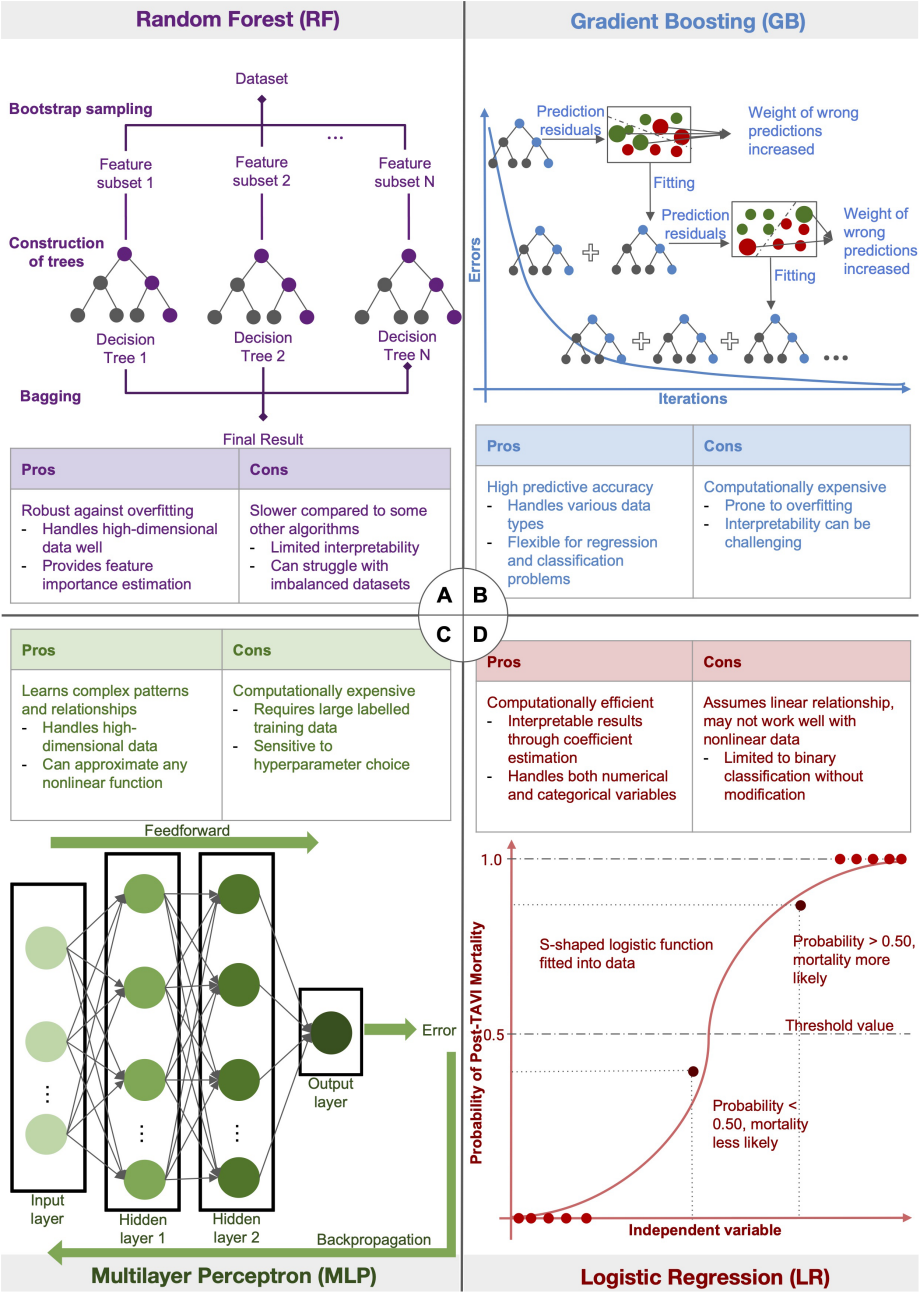
Diagram illustrating the different AI algorithms: figure summarising four of the different AI algorithms used in the included studies. (**A**) The random forest model first divides the dataset into subsets using bootstrap sampling, then generates different decision trees from the same subset. The trees are then averaged to generate the final decision tree. (**B**) Gradient Boosting uses a stage-wise progression to combine weak learning models sequentially to produce an ensemble of different models with minimal prediction errors. In each iteration, the weight of wrong predictions is increased in order to improve the learning model in the successive iteration. (**C**) Multilayer Perceptron, a type of artificial neural network, inputs data into at least 3 layers of nodes: an input layer, a hidden layer and an output layer. Backpropogation of data is used for learning to minimise prediction errors. (**D**) Logistic Regression fits a logistic function onto a dataset. Maximum likelihood estimation is used to produce a curve with maximum likelihood.

A summary of the different AI algorithms, as well as their pros and cons, is provided in Figure 2. The RF model, which divides the dataset into subgroups using bootstrap sampling and produces numerous decision trees from the same subset, is one of the AI techniques utilized in the included studies. The final decision tree is then produced by combining these trees. GB, another used approach, sequentially merges weak learning models in a step-wise process. By raising the weight of incorrect predictions in each iteration to enhance succeeding models, the goal is to produce an ensemble of models with a minimum amount of prediction errors. An artificial neural network, specifically an MLP has at least three layers of nodes: an input layer, a hidden layer, and an output layer. Backpropagation is used to change weights and reduce prediction errors throughout the learning phase after data is fed into the network. Lastly, LR adapts a logistic function to the dataset. The likelihood of the observed data is maximized by using maximum likelihood estimate to draw the curve. For binary classification tasks, this approach is frequently employed. Further elaboration on the different AI algorithms can be found in the Supplementary materials and individual AI methods are also categorized in the Supplementary Table S2.

### Meta-analysis of mortality

All 10 studies were subjected to meta-analysis. 5 studies (29, 32–34, 38) that reported data comparing the predictive ability of AI with traditional clinical risk scores were included in a two-arm meta-analysis. The results from all 10 studies were pooled in a single-arm meta-analysis.

#### Meta-analysis comparing predictive ability of AI vs. traditional scores

**Figure 3:**
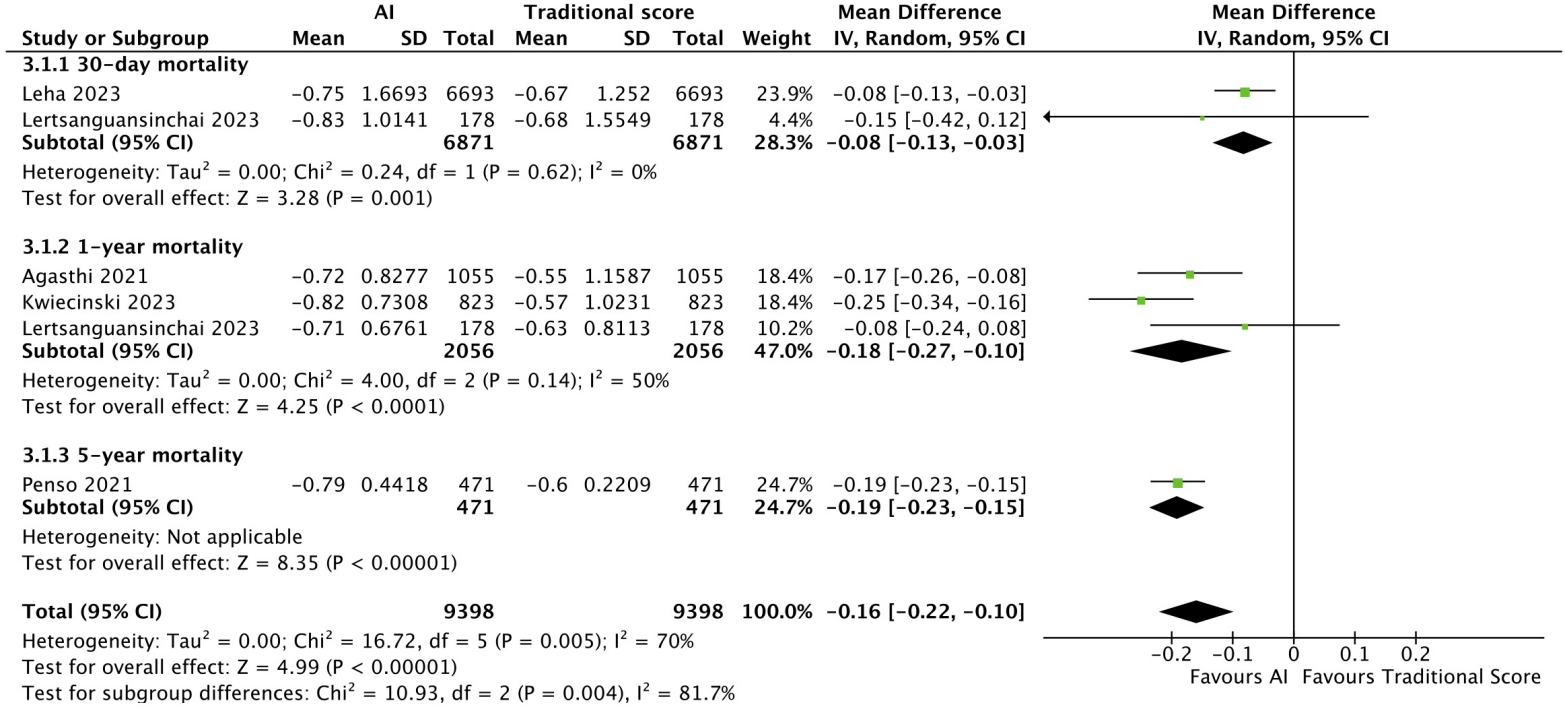
Forest plot comparing AI and traditional scores: forest plot comparing mean AUC values of AI mortality predictions to that of traditional scores. Subgroup analysis was performed, dividing 5 studies into 3.1.1 30-day mortality, 3.1.2 1-year mortality and 3.1.3 5-year mortality, with AI showing an overall better performance than traditional scores.

From our pooled analysis of 5 studies and a total of 9,398 patients, we observed a significantly higher mean AUC in cohorts where post-TAVI mortality was predicted with AI than when traditional scores were used on the same population (MD: −0.16, CI: −0.22 to −0.10, *p *< 0.00001, I2: 70%). 30-day mortality subgroup analysis of 2 studies (6,871 patients) (33, 34) also showed a significantly higher mean AUC with AI predictions than with traditional score predictions (MD: −0.08, CI: −0.13 to −0.03, *p *= 0.001, I2: 0%). Similar findings were observed in the 1-year mortality subgroup, consisting of 2 studies (2,056 patients) (29, 32), with AI showing an overall better performance than traditional scores (MD: −0.18, CI: −0.27 to −0.10, *p *< 0.0001, I2: 50%). (Figure 3.).

#### Meta-analysis of pooled AUC means of 10 studies

**Figure 4:**
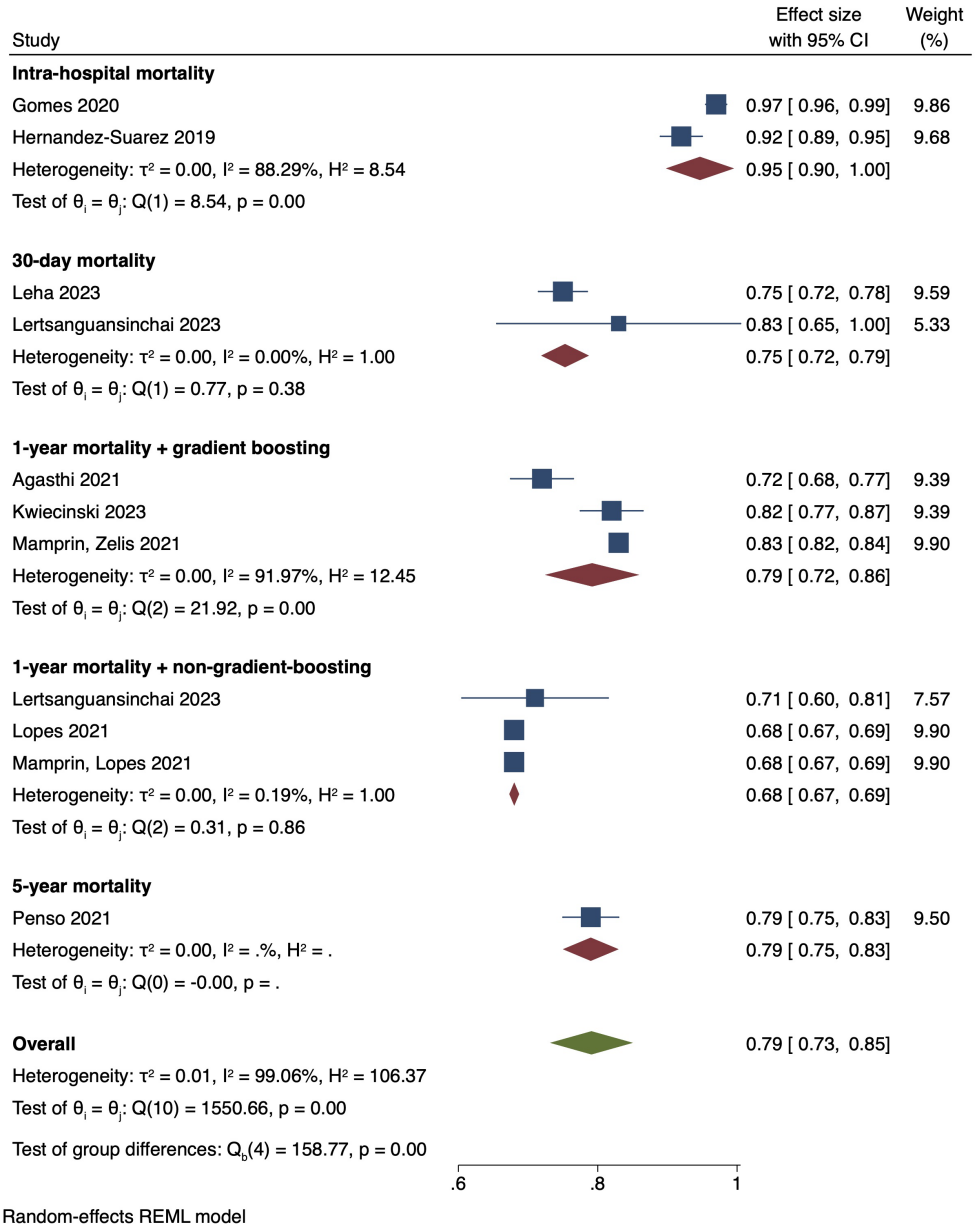
Pooled mean AUC of included studies: forest plot of pooled mean AUC values for AI-predicted post-TAVI mortality, with intra-hospital, 30-day, 1-year and 5-year mortality subgroups.

**Figure 5:**
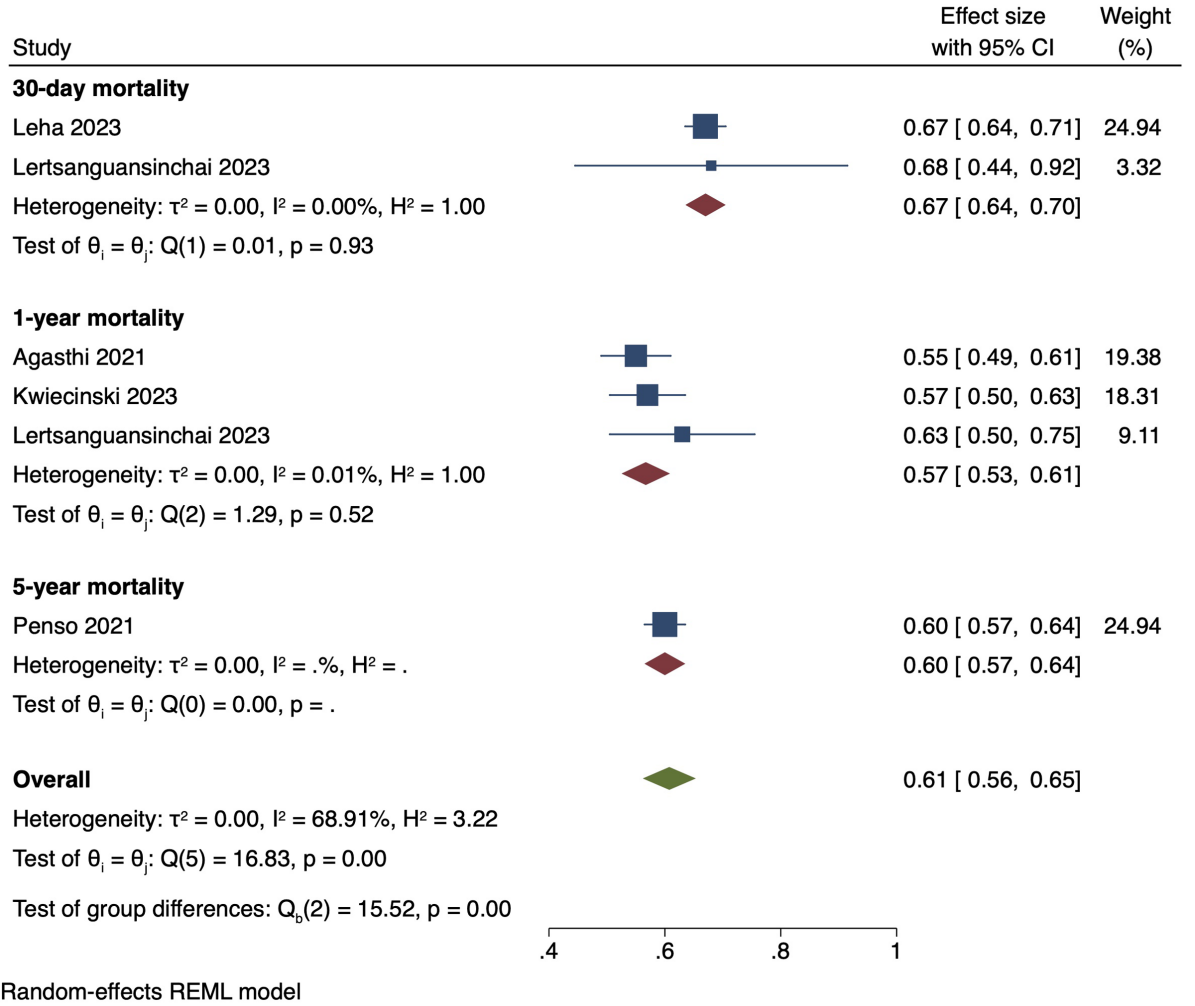
Pooled mean AUC of traditional risk scores: forest plot of pooled mean AUC values for traditional risk score-predicted post-TAVI mortality, with intra-hospital, 30-day, 1-year and 5-year mortality subgroups.

The single-arm meta-analysis of all 10 included studies, and a combined cohort of 22,933 patients, showed a pooled mean AUC of 0.79 [0.73, 0.85]. Due to the high overall heterogeneity (I2 = 99.06%), a subgroup analysis was conducted, separating the studies into intra-hospital mortality, 30-day mortality, 1-year mortality with gradient boosting, 1-year mortality with non-gradient-boosting and 5-year mortality. The pooled mean AUC values of 2 studies reporting intra-hospital mortality was 0.95 [0.90, 1.00], I2: 88.29%. 2 studies with 30-day mortality outcomes had a mean AUC value of 0.75 [0.72, 0.79], I2: 0%. For the 1-year mortality subgroup, 3 studies featuring the use of gradient boosting algorithms had a pooled AUC of 0.79 [0.72, 0.86], I2: 91.97%, while that of the 2 studies that used non-gradient-boosting models was 0.68 [0.67, 0.69], I2:0.19%. For 5-year mortality, 1 study reported an AUC of 0.79 [0.75, 0.83]. Even after subgroup analysis the heterogeneities of the intra-hospital and 1-year mortality with gradient boosting subgroups were still high (Figure 4). This was likely due to the differing parameters used to train the AI models, discordant datasets, and/or the fact that AI model performance varies with the training dataset. A further elaboration on the heterogeneity can be found in the discussion section. Finally, the results of our single-arm meta-analysis of traditional risk scores reported in 5 studies demonstrated a pooled mean AUC value of 0.61 [0.56, 0.65]. The traditional risk score AUC for 30-day and 1-year mortality AUC was 0.67 [0.64, 0.70] and 0.57 [0.53, 0.61], respectively. For 5-year mortality, the traditional risk score AUC reported in 1 study was 0.60 [0.57, 0.64] (Figure 5).

## Discussion

To the best of our knowledge, this is the first meta-analysis in literature comparing AI models against traditional scores in predicting post-TAVI mortality. Overall, the results of our double-arm meta-analysis demonstrate that applying AI to predict death in TAVI cases has a higher predictive accuracy than conventional clinical scoring techniques. In particular, the 30-day (AUC: 0.75) and 1-year mortality (AUC: 0.79) with non-gradient-boosting algorithm subgroups both demonstrated high mean AUC values with low heterogeneity. In our combined analysis of traditional risk scores, the pooled mean AUC value for intra-hospital mortality, 30-day mortality, 1-year mortality with gradient boosting, 1-year mortality without gradient boosting, and 5-year mortality was 0.95 [0.90, 1.00] 0.75 [0.72, 0.79], 0.79 [0.72, 0.86], 0.68 [0.67, 0.69] and 0.79 [0.75, 0.83], respectively. Hence from gross comparison of short- and long-term AUC alone, AI-based models may fare better than current scoring methods at correctly predicting death outcomes in TAVI patients. This suggests that AI models have the potential to aid clinicians in predicting post-TAVI mortality in patients and act as an adjunct or alternative to traditional clinical scores. AI models are able to process large amounts of diverse patient data and can be modelled to continuously analyse new data in order to make accurate predictions in real time. This is a significant advantage over traditional scores, which can only utilise a limited number of variables to predict outcomes.

In the study by Kwiecinski et al. (29), the number of packed red blood cell units transfused, length of hospital stay and minimum estimated glomerular filtration rate provided the greatest contribution to the AI model, with specificities of 94 (92–97)%, 33 (29–37)% and 53 (49–58)%, respectively (29). No other studies reported the specificity values for the individual clinical variables used in their predictive models. As an illustration of the multiplicity of variables that AI models can evaluate, the main 30-day mortality predictive variables reported in the study by Lertsanguansinchai et al. (31) were height, chronic lung disease, STS score, preoperative left ventricular ejection fraction (LVEF), age, and preoperative left ventricular outflow tract velocity time integral (VOT VTI), while the main 1-year mortality variables were preoperative LVEF, STS score, hear rate, systolic blood pressure, home oxygen use, serum creatinine level, and preoperative LVOT Vmax. A more detailed summary of the variables used across the included studies can be found in Supplementary Table S1.

Researchers often compare the AI-generated death predictions with the actual mortality results seen in real-world TAVI patients to assess model accuracy. This evaluation aids in determining the degree to which the AI models are capable of making trustworthy and precise forecasts of post-operative mortality. Here, we provide a brief summary of the advantages and disadvantages associated with each AI algorithm featured in our study.

Firstly, the RF model is capable of analysing high-dimensional data with a huge number of diverse predictors, which can even exceed the number of observations (39).While a single decision tree is prone to noise and overfitting when grown on its training set, the RF model improves accuracy by averaging multiple decision trees. However, due to their complexity, RF models tend to have low intrinsic interpretability (40). Additionally, the presence of dependent observations in data may contribute to increased bias and inaccurate predictive variable selection (41). While the GB algorithm minimises errors and maximises predictive accuracy in the final model, GB algorithms, like the RF models, may also suffer from low interpretability. The GB model may also be prone to over-fitting if the additive process of gradient boosting is not regularised. ANN and MLP algorithms are suitable for analysing non-linear relationships between dependent and independent variables. However, these models are difficult to apply to real-time predictions and are also prone to overfitting (42). Finally, LR algorithms can only be used to predict discrete functions and cannot predict continuous outcomes. It also assumes a linear relationship between dependent and independent variables.

Overall, AI-based prediction models are able to take into account a myriad of patient data to generate more accurate predictions on post-TAVI mortality than traditional scores. However, such models require a large number of patient variables to generate predictions, some of which may not be readily available to clinicians in the immediate clinical setting. Hence, at present, AI-based prediction models may be less user-friendly than simple traditional risk scores. In future, further research into simpler AI-based models that are able to use easily-available clinical parameters in predictions is needed to increase the clinical utility of these models.

Firstly, the RF model constructs a series of decision trees and utilises bagging and randomisation of predictors in order to accurately predict outcomes (24). The original dataset is first divided into smaller subsets through random feature selection. Individual decision trees are then grown on the subsets, allowing for the construction of many decision trees with low correlation to one another. Finally, multiple decision trees are averaged, thereby minimising variance and improving the accuracy of the final predictive model (24). The RF model is thus capable of analysing high-dimensional data with a huge number of diverse predictors, which can even exceed the number of observations (39).

While a single decision tree is prone to noise and overfitting when grown on its training set, the RF model improves accuracy by averaging multiple decision trees. However, due to their complexity, RF models tend to have low intrinsic interpretability (40). Additionally, the presence of dependent observations in data may contribute to increased bias and inaccurate predictive variable selection (41).

The GB model involves an ensemble of weak learner decision trees. Each weak learner progresses through a stepwise progression, where previous iterations of weak learners are combined into a successive strong learner, correcting for the errors of the preceding learner each time (25, 26). Eventually, this process is repeated until errors are minimised and predictive accuracy maximised in the final model. However, similar to the RF model, GB algorithms may also suffer from low interpretability. The GB model may also be prone to over-fitting if the additive process of gradient boosting is not regularised.

The ANN algorithm is a mathematical model developed based on the transmission of signals amoung neural networks in biological nervous systems. An input signal is channelled through a collection of nodes, which are artificial neurons (27). Each node analyses the input signal and then transmits an output to each of its connected neurons, mimicking the transmission of action potentials across neurons and synapses in the human brain. The nodes are also organised into layers. The input signal travels from the first input layer to the last output layer, undergoing different transformations at each layer.

The MLP algorithm is a class of feedforward ANN that is comprised of an input layer, one or more hidden layers and an output layer. The input signal first passes sequentially through the layers in a feedforward process until an output is generated. Subsequently, the data is then backpropagated from the output layer back into the hidden layers. The error signal of each node that contributed to the overall error is determined and the weights of the network updated until the gradient of the mean squared error converges. While ANN and MLP are able to analyse non-linear relationships between dependent and independent variables, these models are difficult to apply to real-time predictions and are also prone to overfitting (42).

The LR algorithm fits a logistic function onto a dataset and predicts the probability of an independent variable, such as mortality, from a dependent variable. The maximimum likelihood estimation method is most commonly used to maximise the likelihood function and find the optimal fit of the model (28). LR algorithms can only be used to predict discrete functions and cannot predict continuous outcomes. It also assumes a linear relationship between dependent and independent variables.

Overall, AI-based prediction models are able to take into account a myriad of patient data to generate more accurate predictions on post-TAVI mortality than traditional scores. However, such models require a large number of patient variables to generate predictions, some of which may not be readily available to clinicians in the immediate clinical setting. Hence, at present, AI-based prediction models may be less user-friendly than simple traditional risk scores. In future, further research into simpler AI-based models that are able to use easily-available clinical parameters in predictions is needed to increase the clinical utility of these models.

## Limitations

The main limitations of this study were firstly, the high heterogeneity of the data, specifically in comparing the intra-hospital mortality subgroup, 1-year mortality with gradient boosting subgroup and the overall pooled AUC of all 10 studies. We postulated that this was due to significant differences in the training datasets used for each AI model. Fundamentally, there was large variability in the quantity of data, as well as in the type and number of parameters used to predict mortality in each model. In addition, due to the lack of studies comparing the same AI algorithms and the same control group, it was difficult to perform a more homogenous subgroup analysis on each AI algorithm. Hence, our results may not be generalisable to all AI algorithms or all datasets predicting mortality in patients post-TAVI.

Secondly, all the studies included in our analysis were of a retrospective nature. There was also a lack of data comparing AI model predictive performance to traditional scores. Therefore, further research, including randomized controlled trials, comparing the use of AI algorithms with traditional scores in predicting post-TAVI mortality will be needed in the future in order to determine the validity of our observations.

Finally, our study did not analyse other endpoints that are known post-TAVI complications, such as stroke, pacemaker need and heart failure, and focused solely on mortality. Hence, this may limit the applicability of our findings in clinical practice.

## Conclusion

Personalized risk assessments are the ultimate goal of research into AI systems for predicting patient outcomes in TAVI procedures. The potential of AI-generated mortality forecasts to improve the precision and value of risk assessment in TAVI is highlighted in this systematic review. AI can help healthcare providers predict and monitor patient outcomes, which will hopefully result in better decision-making and more desirable post-TAVI outcomes in future.

## Data Availability

The authors confirm that the data supporting the findings of this study are available within the article and its supplementary materials. Derived data supporting the findings of this study are available from the corresponding author on request.
