# Supplementary material for: Harnessing the power of artificial intelligence in predicting all-cause mortality in transcatheter aortic valve replacement: a systematic review and meta-analysis
Source: Front Cardiovasc Med. 2024 May 31;11:1343210. doi: 10.3389/fcvm.2024.1343210 (PMC11176615; doi:10.3389/fcvm.2024.1343210)
Supplement: Supplementary file 1 [file Datasheet1.pdf]

Supplementary Material

Use of AI algorithms

This part of the paper investigates the working principles, advantages, and disadvantages of the top-performing AI methods mentioned in 10 previously referenced studies. Our analysis focuses on assessing the suitability of these algorithms in terms of accuracy, interpretability, and ease of implementation.

Logistic Regression is a method that estimates the probability of an outcome by applying the logistic function to a linear combination of input features. Its computational efficiency and ability to provide interpretable results through coefficient estimation stand out. However, Logistic Regression assumes a linear relationship, which may limit its performance with nonlinear data.

The Multilayer Perceptron, a feedforward artificial neural network model, is known for its capability to approximate complex input-output relationships. It utilizes interconnected artificial neurons and nonlinear activation functions. While it can handle high-dimensional data and learn intricate patterns and relationships, the Multilayer Perceptron is computationally expensive, requires a large labelled training dataset, and is sensitive to hyperparameter choice.

Decision Trees offer a hierarchical structure of if-else conditions to partition the input space and predict outcomes through majority voting. They are characterized by their ease of understanding, ability to handle numerical and categorical data, and capacity to capture nonlinear relationships and interactions. However, Decision Trees are prone to overfitting, sensitive to small changes in the data, and biased towards features with more levels or categories.

Gradient Boosting is an ensemble method that combines weak predictive models, often decision trees, to create a more accurate model through iterative model fitting. It is highly regarded for its high predictive accuracy, ability to handle various data types, and flexibility for both regression and classification problems. However, Gradient Boosting algorithms can be computationally expensive, prone to overfitting, and may present challenges in terms of interpretability.

Random Forest, another ensemble method, constructs multiple decision trees using random feature subsets and aggregates their predictions. It excels in handling high-dimensional data, provides estimates of feature importance, and offers robustness against overfitting. However, Random Forest is generally slower compared to some other algorithms, has limited interpretability, and may face difficulties when dealing with imbalanced datasets.

Gradient Boosting on Decision Trees is a powerful ensemble model that sequentially adds decision trees to correct mistakes made by previous trees. It can capture complex nonlinear relationships and handle missing data and mixed feature types. However, it carries the risk of overfitting, is computationally expensive, and may pose challenges in terms of interpretability.

Extreme Gradient Boosting is an optimized gradient boosting framework that employs regularization techniques to control overfitting. It achieves high predictive accuracy, handles

missing values, and allows for feature selection. Nonetheless, Extreme Gradient Boosting requires complex hyperparameter tuning, can be computationally expensive, and may present challenges in terms of interpretability.

In conclusion, when considering accuracy, interpretability, and ease of implementation, Logistic Regression and Decision Trees emerge as favourable options. Logistic Regression is computationally efficient, provides interpretable results, and performs well with approximately linear relationships. Decision Trees strike a balance between accuracy and interpretability, effectively capturing nonlinear relationships while remaining easy to understand and implement. However, the best algorithm choice depends on the study's specific needs and constraints. Researchers should carefully consider the trade-offs and select the algorithm that aligns best with their study objectives and available resources. Ensemble methods like Gradient Boosting and Random Forest may be more suitable when high predictive accuracy is of utmost importance, despite their potential drawbacks in interpretability and computational requirements. A summary of all these methods is provided in Supplementary Table 2.

Supplementary Table 1. Diagram Illustrating the Different AI Algorithms

Study	No. of variables	Predictors with highest relative influence
**Agasthi 2021 [26]**	163	Lower CPI, lower pre-procedural haemoglobin, lower systolic blood pressure, greater INR, lower diastolic blood pressure, lower BMI, higher aortic valve calcium score, higher pre-procedural creatinine, greater aortic annulus area and lower baseline serum albumin
**Gomes 2020 [27]**	83	Baseline troponin T, peak leukocyte count, septum thickness (Echo), peak creatinine value, left atrium diameter, fever post TAVI, pericardial effusion, peak CRP value, fluoroscopy time, contrast agent use
**Hernandez-Suarez 2019 [28]**	43	AKI, cardiogenic shock, fluid and electrolyte disorders, cardiac arrest, sepsis, dyslipidaemia, hypertension, coagulopathy, current smoking, vascular complications
**Kwiecinski 2023 [29]**	59	Packed red blood cell units transfused, hospital length of stay, minimum eGFR, EuroSCORE II, baseline creatinine, BMI, baseline platelets, baseline LVEF
**Leha 2023 [29]**	155	Duration of intervention, duration of fluoroscopy, serum creatinine, pmax, weight, age, dose-area-product, pmean, amount of contrast, height, LVEF, PA pressure
**Lertsanguansinchai 2023 [31]**	Not reported	Height, chronic lung disease, STS score, preoperative LVEF, age, preoperative LVOT VTI
**Lopes 2021 [32]**	Not reported	Not reported
**Mamprin, Lopes 2021 [33]**	16	Not reported
**Mamprin, Zelis 2021 [34]**	13	AV regurgitation, AV peak gradient, atrioventricular block, beta blockers, BMI, creatinine, general health score, haematocrit, haemoglobin, month of post-procedure recovery, previous devices, QRS duration, smoking status
**Penso 2021 [35]**	83	MR aetiology, stroke volume index, interventricular septal thickness, left atrium area, aortic valve area, mean aortic pressure gradient, creatinine, alanine aminotransferase, haemoglobin, INR, age, spironolactone, angina, EuroSCORE II

AKI: acute kidney injury, AV: aortic valve, BMI: body mass index, CPI: cardiac power index, CRP: c-reactive protein, Echo: echocardiography, eGFR: estimated glomerular filtration rate, INR: international normalised ratio, LVEF: left ventricular ejection fraction, LVOT VTI: left ventricular outflow tract velocity time integral, MR: mitral valve regurgitation, PA pressure: pulmonary artery pressure, pmax: peak AV gradient, pmean: mean AV gradient, STS: Society of Thoracic Surgeons, TAVI: transcatheter aortic valve implantation.

Supplementary Table 2. Diagram Illustrating the Different AI Algorithms

Method	Description	Graphical Visualization	Advantages	Disadvantages
Logistic Regression	Estimates the probability of the outcome by applying the logistic function to a linear combination of input features. It models the relationship between predictors and the outcome.	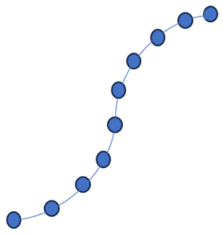	Computationally efficient<br>- Interpretable results through coefficient estimation<br>- Handles both numerical and categorical variables	Assumes linear relationship, may not work well with nonlinear data<br>- Limited to binary classification without modification
Multilayer Perceptron	Feedforward artificial neural network model. It consists of interconnected artificial neurons and uses nonlinear activation functions to approximate complex input-output relationships.	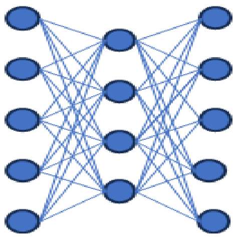	Learns complex patterns and relationships<br>- Handles high-dimensional data<br>- Can approximate any nonlinear function	Computationally expensive<br>- Requires large labelled training data<br>- Sensitive to hyperparameter choice
Decision Tree	Hierarchical structure of if-else conditions. It partitions the input space based on feature values and predicts the outcome by majority voting of training samples within each partition.	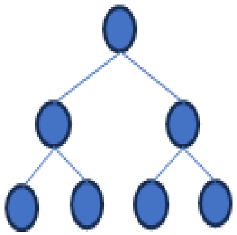	Easy to understand and interpret<br>- Handles numerical and categorical data<br>- Captures nonlinear relationships and interactions	Prone to overfitting<br>- Sensitive to small changes in data<br>- Biased towards features with more levels or categories
Gradient Boosting	Ensemble of weak prediction models. It combines multiple "weak" predictive models, often decision trees, into a single, more accurate model through iterative model fitting.	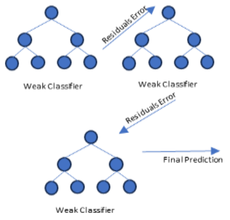	High predictive accuracy<br>- Handles various data types<br>- Flexible for regression and classification problems	Computationally expensive<br>- Prone to overfitting<br>- Interpretability can be challenging

Random Forest	Ensemble of decision trees with random feature subsets. It builds multiple decision trees using random subsets of features and aggregates their predictions for the final outcome.	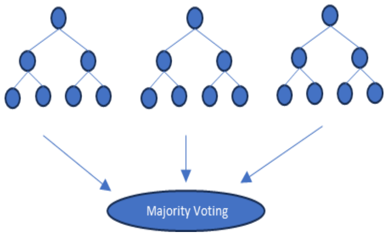	Robust against overfitting<br>- Handles high-dimensional data well<br>- Provides feature importance estimation	Slower compared to some other algorithms<br>- Limited interpretability<br>- Can struggle with imbalanced datasets
Gradient Boosting on Decision Trees	Gradient boosting using decision trees as base models. It sequentially adds decision trees that correct mistakes made by previous trees, resulting in an improved ensemble model.	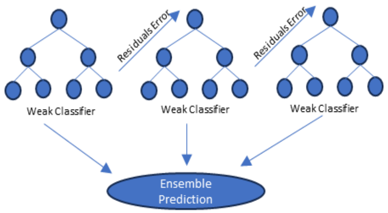	Powerful ensemble model<br>- Captures complex nonlinear relationships and interactions<br>- Handles missing data and mixed feature types	Potential for overfitting<br>- Computationally expensive<br>- Interpretability can be challenging
Extreme Gradient Boosting	Optimized gradient boosting framework. It uses a regularized model formalization and gradient-based optimization to minimize the loss function and control overfitting.	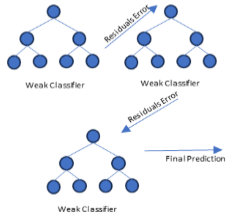	High predictive accuracy<br>- Regularization techniques for overfitting control<br>- Handles missing values and feature selection	Complex hyperparameter tuning<br>- Computationally expensive<br>- Interpretability can be challenging

Supplementary Table 2: AI algorithms commonly employed in related studies *with an overview of their working principles*, graphical visualization, *advantages, and disadvantages***.**
