# Supplementary material for: Harnessing the power of artificial intelligence in predicting all-cause mortality in transcatheter aortic valve replacement: a systematic review and meta-analysis
Source: Front Cardiovasc Med. 2024 May 31;11:1343210. doi: 10.3389/fcvm.2024.1343210 (PMC11176615; doi:10.3389/fcvm.2024.1343210)
Supplement: Supplementary file 2 [file Image1.pdf]

Predictive Analysis of All-Cause Mortality in Transcatheter Aortic Valve Replacement: Harnessing the Power of Artificial Intelligence

What is the role of AI in predicting post-TAVI mortality?

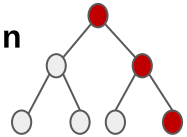

Systematic search using AI algorithms to predict mortality in TAVI patients across **4 databases**

- 10 studies, 22933 patients
- **Pooled AUC: 0.79 [0.74, 0.84]**

- 5 studies, 9398 patients
- **AI algorithms** predict post-TAVI mortality with **higher accuracy** than traditional scores

AI algorithms

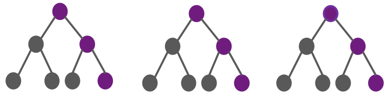

- **Random Forest**: Ensemble of decision trees with random feature subsets aggregated for final outcome

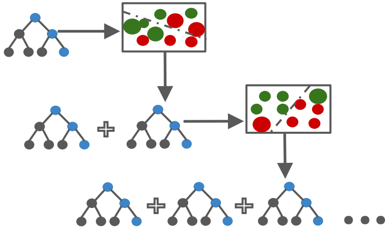

- **Gradient Boosting**: Ensemble of weak prediction models combined into a single more accurate model

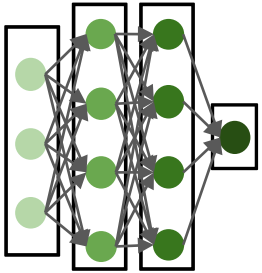

- **Multilayer Perceptron**: Feedforward artificial neural network model, consisting of interconnected artificial neurons

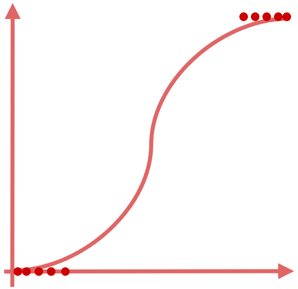

- **Logistic Regression**: Estimates probability of outcome by applying a logistic function to a linear combination of input features
